# Mass Homozygotes Accumulation in the NCI-60 Cancer Cell Lines As Compared to HapMap Trios, and Relation to Fragile Site Location

**DOI:** 10.1371/journal.pone.0031628

**Published:** 2012-02-09

**Authors:** Xiaoyang Ruan, Jean-Pierre A. Kocher, Yves Pommier, Hongfang Liu, William C. Reinhold

**Affiliations:** 1 Department of Health Sciences Research, Mayo Clinic College of Medicine, Rochester, Minnesota, United States of America; 2 Laboratory of Molecular Pharmacology, National Cancer Institute, Bethesda, Maryland, United States of America; Duke-National University of Singapore Graduate Medical School, Singapore

## Abstract

Runs of homozygosity (ROH) represents extended length of homozygotes on a long genomic distance. In oncology, it is known as loss of heterozygosity (LOH) if identified exclusively in cancer cell rather than in matched control cell. Studies have identified several genomic regions which show consistent ROH in different kinds of carcinoma. To query whether this consistency can be observed on broader spectrum, both in more cancer types and in wider genomic regions, we investigated ROH patterns in the National Cancer Institute 60 cancer cell line panel (NCI-60) and HapMap Caucasian healthy trio families. Using results from Affymetrix 500 K SNP arrays, we report a genome wide significant association of ROH regions between the NCI-60 and HapMap samples, with much a higher level of ROH (11 fold) in the cancer cell lines. Analysis shows that more severe ROH found in cancer cells appears to be the extension of existing ROH in healthy state. In the HapMap trios, the adult subgroup had a slightly but significantly higher level (1.02 fold) of ROH than did the young subgroup. For several ROH regions we observed the co-occurrence of fragile sites (FRAs). However, FRA on the genome wide level does not show a clear relationship with ROH regions.

## Introduction

In diploid organisms, SNPs are categorized into heterozygotes and homozygotes according to the difference/identity between paternal and maternal alleles. Loss of heterozygosity (LOH) [1] in a cell represents the conditions of loss of normal function of one allele when the other allele was already inactivated. LOH may cause tumor suppressor gene loss of function, which is associated with oncogenesis [2].

Genomic regions affected by LOH often exhibit an extended length of homozygous SNPs, which forms regions with low diversity between two copies of DNA. Studies in *E. coli* [3], *Trypanosoma brucei* [4] and mouse [5] show the importance of extended sequence similarity/diversity in promoting/preventing homologous recombination. This recombination, if happened during mitotic process, maybe related to subsequent LOH [6] and affects genome stability [7], [8]. Study by Bacolod, M.D. [9] shows that colorectal cancer patients have average length of identical by descent region twice the length of healthy population. Based on the colorectal cancer data, they later proposed a cancer gene activity model (CGAM) [10] in an effort to explain how autozygosity influences cancer predisposition. Similarly, a study by Assie, G *et al.* [11] investigated breast, prostate, head and neck carcinomas and found 16 common loci that have significantly increased germline homozygosity frequency.

From these previous works, we conclude that 1) LOH is related to diseases and 2) in cancer there may be an extension of germline homozygous region. This has been shown population wise in some type of cancer, and in certain loci across three different types of carcinoma. Question remains if this phenomenon can be extrapolated to multiple cancers at the whole genome scale. We are hypothesizing that the increased size of the homozygous regions in cancer arises from the extension of regions found in germline. We are also hypothesizing that the genomic position of homozygous regions are not equally distributed across the genome (conserved across individuals) and therefore could depend on the local properties of the genomic DNA. Finally, we are postulating that homozygous regions in germline that are dramatically extended in cancer could have the natural propensity to expend upon aging. In this manuscript we will refer to these homozygous regions as runs of homozygosity (ROH).

We have performed a genome wide comparison of ROH position and length in cancer cell lines from the NCI-60 library as well as in germline samples from young and adult individuals obtained from 30 HapMap Caucasian trio families. We also investigated if a proximity relationship exists between ROH positions and fragile sites (FRA) that undergo more often DNA damage repairs. In addition we investigated similar relationship between ROH and microRNA (miRNA) sites that have been shown to be associated to FRA regions [12].

## Materials and Methods

### NCI-60 Cancer Cell Line and HapMap Samples

The NCI-60 was developed as an anticancer drug screen panel by the US National Cancer Institute (NCI), Developmental Therapeutics Program (DTP, http://dtp.nci.nih.gov/) [13]. It contains 60 Caucasian tumor cell lines from brain (BR), central nervous system (CNS), colon (CO), lung (LC), white blood cell (LE), melanocyte (ME), ovary (OV), prostate (PR) and renal (RE). Detailed information about NCI-60 is available at CellMiner website (http://discover.nci.nih.gov). To prepare cell lines for Affymetrix 500 K SNP array analysis, frozen stocks of the NCI-60 were obtained from the NCI Developmental Therapeutics program (NCI DTP). The cells were cultured as described previously [14], and then thawed, placed in RPMI 1640 (Lonza Walkersville, Inc.) containing 5% fetal calf serum (Atlantic Biologicals) and 2 mM glutamine (Invitrogen Corporation). For compatibility with our other profiling studies, we used the same batch of serum used by DTP, and the procedures were done or overseen by the same researcher (WCR). The colon cell line HT29 was removed due to contamination. Results of two replicates on ovary cell line OVCAR-3 were merged, and discrepant results were set to unknown. After exclusion and merge, SNP array data of 59 cancer cell lines were used in the final analysis. The NCI-60 Affymetrix 500 K SNP array data is publicly available from Gene Expression Omnibus (GSE32264).

The International HapMap Project is a muti-country effort to identify and catalog genetic similarities and differences in human beings (http://HapMap.ncbi.nlm.nih.gov/). Only HapMap samples with European origin were used to minimize the ethnic difference with the NCI-60 samples. A total of 59 cancer cell lines and 30 normal HapMap CEU trio families (30 children (young) +60 parents (adult)) genotyped with the Affymetrix 500 K SNP array were analyzed. Genotype calls were made using Affymetrix Power Tool (APT) (Linux, version 1.12.0) using the BRLMM algorithm (http://media.affymetrix.com/support/technical/whitepapers/brlmm_whitepaper.pdf) at the default confidence level (< = 0.5). The average “NoCall” rate is 4.2% in the NCI-60 and 0.5% in HapMap samples.

### Identify Runs of homozygosity (ROH), ROH frequency (ROHF) and Hemizygous deletion frequency (HDF)

Existing studies primarily identify ROH by moving a fixed-size window along SNP genotypes and detecting long stretch of homozygotes. Drawbacks of this scheme are 1) there is no standardized criteria for calling ROH, 2) different settings on window size, homozygous SNP number, accommodation for genotyping error, or homozygotes stretch length threshold may significantly influence the result [15]. To avoid bias that may arise from inappropriately chosen criteria, ROH in the current study was primarily detected by basic Hidden-Markov Model (HMM) procedure proposed by Beroukhim R. *et al.* [16]. While still arbitrary, HMM is less sensitive to interspersed heterozygotes in that it tracks the state change between low and high heterozygosity rates. Although HMM was primarily used to detect LOH, its working principle makes it applicable to ROH detection. By locating the position where transition probability is higher than a given threshold, HMM differentiates ROH from normal heterozygosity regions. Basic-HMM procedure available in the dChip software (Version 2010/01) [17] was used to perform the analysis with default settings. SNPs annotations from Human genome build 19 were used. In addition to HMM algorithm, we also used PLINK (Version 1.07) (http://pngu.mgh.harvard.edu/purcell/plink/) [18] (based on fixed size window) as an alternative way to detect ROH. The parameters used in PLINK are provided as (Table S5).

ROH frequency (ROHF) is defined as the proportion of samples with ROH for each SNP locus. To reduce background noise in the ROHF calculation, ROHF in FRA regions is calculated by taking mean values of the upper 95^th^ percentile ROHF in the ±5 Mb range.

Copy number variation was computed by pennCNV [19], which provides integer copy number estimate. The copy number information was used to calculate hemizygous deletion frequency (HDF) that is defined as the proportion of samples with only 1 copy of allele for each SNP locus. The average number of SNP with hemizygous deletion is about 25,000 (5% SNPs on 500 K array). The maximum HDF is 0.24 in the NCI-60 cell lines.

### miRNA and FRA database

**Figure 1:**
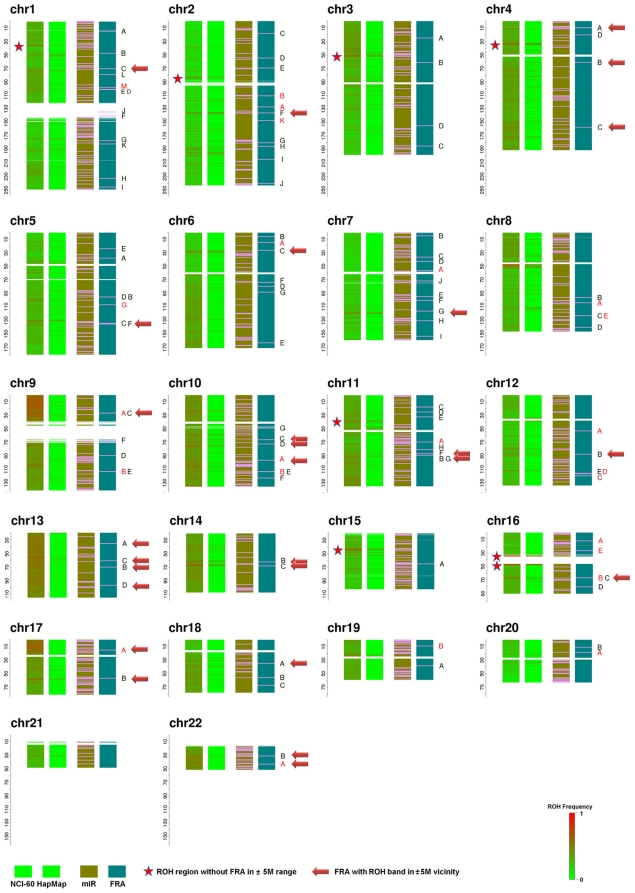
NCI-60 and HapMap ROH pattern, and miRNA, FRA position. Red arrows on the right indicate the FRAs with average upper 95^th^ percentile ROHF larger than 0.5 in a ±5 Mb vicinity. Red asterisks indicate high ROHF bands (average upper 95^th^ percentile ROHF>0.6) without FRA in ±5 Mb vicinity. Rare FRAs are marked by red color. The gaps between the sections of the chromosomes (for example at 130 Mb nucleotides in chromosome 1) contain the centromere. The top portion corresponds to *p* arm, and bottom portion corresponds to *q* arm.

Information on 1049 miRNA was obtained from miRBase www.miRNAbase.org (Version 16) [20]. After excluding records located on chromosome X and Y, 955 miRNA records were involved in current analysis. FRA information was obtained from the HUGO Gene Nomenclature Committee (HGNC) www.genenames.org/cgi-bin/hgnc_stats.pl (Last Update 09/11/10). Totally 111 FRAs located on autosomes were involved in current analysis. Since only FRAs' cytogenetic position is available, we converted these positions in genomic coordinates as follow. A database covered 42,574 gene records (obtained from NCBI Entrez Gene database) with both cytogenetic and genomic location was used to map the genomic location of FRAs. As a result, minimum genomic start and maximum genomic end positions were used to represent the FRA region, and the mean value was used to represent the FRA location (As indicated on the 4^th^ bar in Figure 1). For each FRA, we considered, based on a recombination rate less than 0.5, that ±5 Mb can be used as a maximum distance in our search for FRA related genomic changes. We also reduced the region down to ±1 Mb when analyzing miRNA-FRA relationship.

### Statistical Analysis

ROHF correlation coefficients (Table 1 12^th^ column) between NCI-60 and HapMap samples were obtained after adjusting for SNP density and HDF. Correlation P values were further adjusted by Bonferroni correction.

**Table 1:** Average ROHF on all chromosomes in NCI60 and HapMap samples.

	NCI60					HapMap						
Chr	p+q^a^	p	q	FRA corr^b^	p(FRA corr)	p+q	p	q	FRA corr	p(FRA corr)	Corr^c^	SNP number
1	0.27	0.31	0.24	0.14	0.00E+00	0.04	0.03	0.04	0.07	0.0E+00	0.33	40200
2	0.18	0.14	0.20	0.00	7.30E-01	0.04	0.03	0.05	0.02	1.0E+00	0.73	41376
3	0.30	0.35	0.24	−0.02	1.50E-04	0.03	0.03	0.03	−0.17	1.2E-217	0.44	33761
4	0.36	0.34	0.36	−0.08	1.39E-43	0.04	0.04	0.04	−0.03	3.4E-04	0.72	32321
5	0.27	0.23	0.28	0.24	0.00E+00	0.03	0.03	0.03	0.21	0.0E+00	0.65	32039
6	0.30	0.20	0.36	−0.17	6.26E-204	0.04	0.04	0.03	−0.01	1.0E+00	0.31	31384
7	0.20	0.17	0.22	−0.06	3.45E-23	0.03	0.02	0.03	0.02	1.0E+00	0.65	25790
8	0.31	0.36	0.28	−0.25	0.00E+00	0.05	0.04	0.05	−0.22	1.0E-293	0.56	27446
9	0.49	*0.60*	0.42	−0.19	1.47E-190	0.03	0.03	0.03	−0.01	1.0E+00	0.20	22862
10	0.43	0.43	0.43	−0.01	3.52E-02	0.04	0.03	0.05	0.04	1.3E-09	0.66	28476
11	0.37	0.35	0.38	−0.28	0.00E+00	0.04	0.05	0.04	−0.03	2.2E-03	0.43	26241
12	0.26	0.28	0.25	−0.04	1.58E-08	0.03	0.03	0.04	0.14	0.0E+00	0.51	24925
13	0.51	NA^d^	*0.51*	0.18	0.00E+00	0.03	NA	0.03	0.22	0.0E+00	0.44	19106
14	0.40	NA	0.40	0.30	0.00E+00	0.03	NA	0.03	0.38	0.0E+00	0.81	15712
15	0.26	NA	0.26	−0.03	3.48E-04	0.03	NA	0.03	−0.01	1.0E+00	0.74	14339
16	0.30	0.27	0.31	−0.02	2.91E-03	0.03	0.02	0.03	0.01	1.0E+00	0.87	15269
17	0.44	*0.57*	0.39	0.29	0.00E+00	0.02	0.01	0.03	0.08	0.0E+00	0.20	11255
18	0.34	0.24	0.37	0.45	0.00E+00	0.03	0.00	0.03	0.28	0.0E+00	0.56	14873
19	0.34	0.35	0.33	−0.15	2.01E-32	0.02	0.04	0.02	−0.20	7.5E-54	0.79	6394
20	0.18	0.22	0.15	0.41	0.00E+00	0.03	0.03	0.03	−0.01	1.0E+00	0.43	12398
21	0.29	0.18	0.29	NA	NA	0.02	0.00	0.02	NA	NA	0.78	7113
22	0.43	NA	0.43	0.37	0.00E+00	0.01	NA	0.01	0.23	0.0E+00	0.48	6161
All^e^	0.32	0.30	0.32	−0.02	2.2E-16	0.03	0.03	0.04	0.02	2.2E-16	0.33	489441

a **p and q stands for short and long arm, respectively.**

b **Pearson correlation between ROHF and FRA(±5 M).**

c **Pearson correlation between the NCI-60 and HapMap ROHF, all p values less than 1e-10 after controlling for SNP density.**

d **No SNP coverage on Affymetrix 500 k array.**

e **Chromosome X and Y were excluded from analysis.**

Adult and young subgroups are different in sample size (*N*_adult_ = 60, *N*_young_ = 30), thus direct comparison on their ROHF would be inappropriate (consider SNPs where only one adult and zero young has ROH). To eliminate the bias, we shrunk the number of adults to 30 individuals by non-redundant re-sampling. 1000 random adult datasets were generated. ROHF was calculated for each dataset. For each locus the mean value was used to represent the adult ROHF, which was compared with young ROHF to determine statistical significance (Student t-test followed by Bonferroni correction). The null hypothesis is that there is no significant difference in ROHF between adult and young. To estimate the power of the test, we also calculated the proportion of datasets with positive ROHF_diff_ ( = ROHF_adult_−ROHF_young_) among datasets that show significant difference between adult and young.

Pathway analysis was conducted by selecting the SNPs with ROHF difference ranked top 5000 (ROHF_diff_ = 0.59), 10000 (0.54), 15000 (0.51), 20000 (0.49), and 25000 (0.47) between NCI-60 and HapMap sample, as well as between HapMap adult and young subgroups. SNPs selected by applying these different cutoff settings were analyzed with the GeneGO web tool (GeneGO Inc.) to report pathways enriched in significant SNPs.

To avoid the bias caused by sex chromosomes, both chromosome X and Y were excluded from current analysis. All analyses were conducted by using R version 2.10.0 in the Unix environment.

## Results

### ROH in the NCI-60 and HapMap samples

Figure 1 illustrates ROHF among the NCI-60 and HapMap samples, as well as the physical locations of miRNA and FRA sites. The average ROHF is 0.32 for the NCI-60, and 0.03 for the HapMap samples (Table 1). Generally, we observe massive ROH events spread across the whole genome for the NCI-60 cell lines. Chromosomes 9p, 13q, and 17p have the highest levels of ROH, with average ROHF being 0.60, 0.51, and 0.57, respectively. The same chromosome regions with increased ROHF in the NCI-60 were found to occur in the HapMap non-cancerous samples but with lower ROHF (Figure 1). Significant correlations between the NCI-60 and HapMap ROHF were found on all chromosomes before and after adjustment for SNP density and HDF (Table 1, 12^th^ column). Similar patterns were observed using PLINK to compute ROH (data not shown). We also analyzed the HapMap CEPH Affymetrix 100 K array data and found ROHF pattern same as that from 500 K array (Data not shown), indicating that the observed patterns are independent of platform used for genotyping as well as of the application used to compute ROHs. Suspecting that regions with higher level of ROHF in healthy state have stronger ROHF correlation between NCI-60 and HapMap samples, we applied a stepwise increasing cutoff on SNPs according to their ROHF levels in HapMap samples. The results show a stepwise increase in correlation coefficient from 0.34 to 0.5 as the cutoff increases from >0.1 to >0.95 quantile (Figure S1). This indicates that regions with existing ROH in healthy state are more likely to have a higher level of ROHF in cancer cells. We also analyzed the HapMap trios according to their status as adult or young (detailed age information is not available, so only adult/young stratification is used). Both paired and un-paired t test showed significant ROHF differences between adult and young subgroups (p<1.1e-10 after Bonferroni adjustment) with an average of 0.06% higher ROHF in adult subgroup over the whole genome (3.36% in young, 3.42% in adults). Among the 1000 randomly generated adult datasets (following numbers in parenthesis are by PLINK deduction), 662 (833) have ROHF_diff_>0 and 747 (743) have P<10^−7^ (P<0.05 after Bonferroni adjustment, two-tailed student t test). Among the 747 (743) datasets with significance, 552 (686) have ROHF_diff_>0. This equals to a proportion of 73.9% (92.3%).

### ROHF and FRAs

We did not observe a clear relationship between FRA and ROHF on the whole genome scale, They exhibit negative correlation in the NCI-60, and positive correlation in the HapMap samples. Their correlations on different chromosomes also show different directions (Table 1). Despite of this unclear relationship, we indeed observed the co-occurence of several high ROHF bands with FRAs. Among the 111 recognized FRAs, there are 30 bands with an average upper 95^th^ percentile ROHF higher than 0.5 (1.64 standard deviation above mean ROHF) in the vicinity of FRAs (Table 2, 4^th^ column). For these FRAs we see clear ROH bands among the HapMap samples, and also among the NCI-60 samples (with higher ROHFs) at the same physical location. Moreover, 4 FRAs have at least 0.5 ROHF difference between the NCI-60 and HapMap samples (Table 2, 6^th^ column, shown in red). Due to the computational similarity between ROH and LOH, we compared the ROHF with previously reported LOH frequency around FRAs. For FRA16D [21], [22], FRA7G [23] and several other FRAs that were previously reported to have LOH in close vicinity, similar findings were also observed in our data. Cell line specific analysis showed 50% ROHF in renal cancer cell lines at FRA3B, and 57.1% in ovarian at FRA6E, which is close to the reported value of 69% in renal cell carcinoma for FRA3B [24], and 72% in ovarian cancer for FRA6E [25].

**Table 2:** Average upper 95th percentile ROHF and number of miRNA genes around FRAs.

FRA	Chr	CytoPos^a^	ROHFNCI-60^b^	ROHFHapMap^b^	ROHF(NCI-60-HapMap)	NummiRNA (5 M)^c^	NummiRNA (1 M)^c^
FRA1C	1	1p31.2	***0.56***	0.28	0.28	5	0
FRA2F	2	2q21.3	***0.57***	***0.52***	0.05	3	1
FRA4C	4	4q31.1	***0.55***	0.15	0.40	2	0
FRA5C	5	5q31.1	***0.53***	0.36	0.17	4	1
FRA5F	5	5q21	***0.53***	0.34	0.19	5	0
FRA7G	7	7q31.2	***0.56***	0.47	0.09	1	0
FRA9B	9	9q32	***0.51***	0.09	0.42	3	0
FRA9E	9	9q32	***0.51***	0.09	0.42	3	0
FRA9A	9	9p21	***0.75***	0.24	***0.51***	2	0
FRA9C	9	9p21	***0.75***	0.24	***0.51***	2	0
FRA10C	10	10q21	***0.54***	0.22	0.31	2	0
FRA10D	10	10q22.1	***0.56***	0.27	0.29	2	0
FRA10A	10	10q23.3	***0.59***	0.28	0.31	3	0
FRA11B	11	11q23.3	***0.58***	0.27	0.31	3	0
FRA11F	11	11q14.2	***0.58***	0.27	0.31	2	0
FRA11G	11	11q23.3	***0.58***	0.27	0.31	3	0
FRA12B	12	12q21.3	***0.59***	0.37	0.21	0	0
FRA13A	13	13q13.2	***0.56***	0.11	0.45	0	0
FRA13B	13	13q21	***0.58***	0.10	0.48	0	0
FRA13D	13	13q32	***0.59***	0.10	0.49	9	0
FRA13C	13	13q21.2	***0.67***	0.17	***0.50***	1	0
FRA14B	14	14q23	***0.71***	0.47	0.24	2	0
FRA14C	14	14q24.1	***0.74***	***0.65***	0.10	2	0
FRA16B	16	16q22.1	***0.57***	0.29	0.27	5	1
FRA16C	16	16q22.1	***0.57***	0.29	0.27	5	1
FRA17A	17	17p12	***0.61***	0.06	***0.55***	5	0
FRA17B	17	17q23.1	***0.65***	0.30	0.36	6	3
FRA18A	18	18q12.2	***0.55***	0.28	0.27	4	1
FRA22B	22	22q12.2	***0.54***	0.18	0.36	7	2
FRA22A	22	22q13	***0.56***	0.07	0.49	8	1

a **Cytogenetic position.**

b **Average upper 95th percentile ROHF values in corresponding FRA region (Start and end position available in Table S2).**

c **Number of miRNA genes within ±5 Mbs and ±1 Mbs range of FRAs.**

Several high ROHF bands have no recognized FRAs in their vicinity. Table 3 shows a total of eight top ROH bands with average top 95^th^ percentile ROHF ranging from 0.551 to 0.879 but no recognized FRAs nearby. The bands are also marked with red asterisk in Figure 1. Two of these ROH bands are located at the chromosome 16 centromere. A list of genes located in these ROH bands is available in Table S1. On the other hand, we observed that 81 out of 111 FRAs do not have associated significant ROHF elevation in nearby regions (Table S2).

**Table 3:** High ROHF bands without FRA in within ±5 Mb.

Chromosome	Position^a^	Start (Mb)^b^	End (Mb)^b^	ROHF^c^
1p	36030321	35	37	0.608
2p	83276616	82	85	0.551
3p	51216578	50	53	0.666
4p	33354084	32	36	0.726
11p	38675473	37	41	0.561
15q	43729998	42	46	0.66
16p	34923039	34	36	0.879
16q	47735030	46	49	0.755

a **Physical position of SNP locus with maximum ROHF in the range. The middle one was used if multiple SNP loci have equal maximum value.**

b **Approximate start and end position in Mega base pair.**

c **Average upper 95th percentile ROHF in ±5 Mb vicinity around “Position”.**

### ROHF and miRNA

Calin G.A. *et al.* [12] proposed a possible association between FRAs and miRNA location (incidence of 186 miRNA genes within ±1 Mb of FRAs is 13 (ratio = 0.07)). To obtain a comprehensive view of the relationship among FRA, miRNA and ROH banding, we incorporated the miRNA data in this study. Among 955 miRNAs investigated in our study, 63 were within ±1 Mb range of FRA locations (ratio = 0.066). This number increased to 334 after expanding to ±5 Mb range (ratio = 0.35). Pearson correlation analysis shows no association between miRNA location (±1 M range) and ROHF levels (Figure 1, p>0.05 after bonferroni correction). In addition, we investigated the co-occurrence of miRNA and FRA with ROHF elevation. In the ±5 Mb vicinity of FRAs with ROHFs higher than 0.5, the average number of miRNAs is 3.30. This number is 2.94 for FRAs with nearby ROHFs lower than 0.5.

### Pathway Analysis

**Figure 2:**
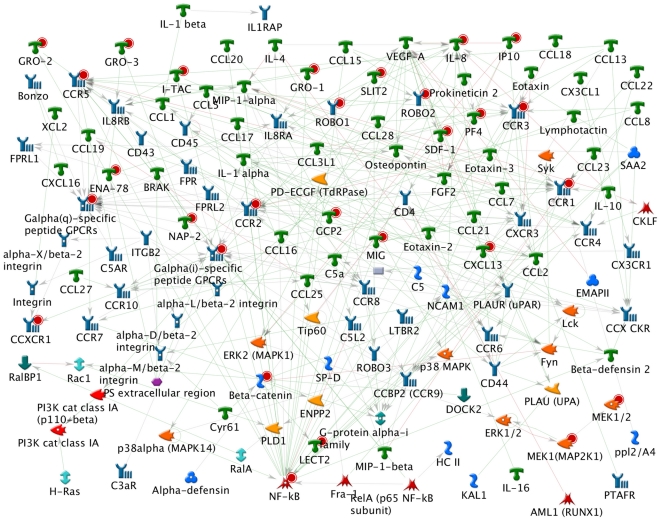
Genes involved in chemotaxis. Pathway analysis on SNPs with top ROHF difference between adult and young subgroups show the involvement of chemotaxis process. Red solid circles show genes covering SNPs with top change (see statistics section for details).

We analyzed the SNPs with the top ROHF differences (See statistical analysis section for detail) between adult and young subgroups, as well as between the NCI-60 and HapMap samples (Figure S2). For the adult versus young subgroup, the top ranked process is the chemotaxis process (Figure 2). The genes present in this category are marked with solid red circles in Figure 2, and tabulated in Table S3. For the NCI-60 versus the HapMap samples, the top ranked process is G1_S cell cycle process (Figure S3 and Table S4), which indicates a change in several known oncogenes such as P53 and LATS2. For these two genes we see ROHF elevations as high as 61% and 50% in the NCI-60 as compared to HapMap samples.

## Discussion

Studies in prokaryotes and eukaryotes [3]–[5] show that high similarity genomic regions may induce homologous recombination, which was proposed as a cause of LOH in mammalian cell [6] and has complex role in genomic stability [7], [8]. In this study, we interrogated the sequence similarity by detecting the ROH status on 500 K array through basic-HMM model as well as PLINK ROH module. Analysis on NCI-60 cancer cell lines and HapMap trios show highly similar ROH patterns (Figure 1), which differ only in strength. The current finding indicates that the massive ROH observed in cancer cells might be an extension of the lower levels ROH observed in healthy cells. We consider the current finding has wide applicability for several reasons. First, the present study is based on 60 different cancer cell lines, which excluded the potential bias caused by the unique genetic construct of a specific type of cancer. Second, rather than using patient-matched control (germ-line) DNA samples obtained from non-tumor tissue, the cases and controls are totally un-matched. Thus their similarity in ROH pattern is not due to the presence of the same genetic background, and may reflect a more general condition of human genome. Finally, the NCI-60 cell lines used for the current SNP array analysis have passage number below 30 [26]. This should minimize the potential effect of culture-induced genomic alteration [27]. On the other hand, we observed that adult subgroup has higher level of ROH than those of young in HapMap Caucasian trios (p<1.1e-10 after the stringent Bonferroni adjustment). Since 1) exactly the same HMM model parameters are used to call ROH independently in adult and young subgroups, and 2) it is groundless to hypothesize that young should born with lower ROH level than adult, we have to accept the alternative hypothesis and propose that an ongoing ROH happened in adults from the time point of their own zygotes formation to the zygotes formation of their child. Worth mentioning is that cell lines rather than original lymphocyte DNA were used in the HapMap SNP array analysis. Thus one can't rule out that the observed difference could be caused by culture-induced mutation or different passage number. However, the current observation is in agreement with reported higher level of LOH in old cells in model organisms [28]. Similar observation was also done in human by Moragoda *et al.* [29] who described age-associated stomach mucosa LOH among healthy people over 60 year of age. Nevertheless, further investigations are required to elucidate whether the ROH level difference observed in our study is resulted from aging, disease, or epigenetic factors.

Our data show some co-occurrence of FRAs and high ROHF band in the same genomic region. This observation is in agreement with previous findings done in some genomics regions [21], [23]. It was proposed that the AT-rich nature of FRA sequences might confer them a highly variable nature [30]–[32]. For some FRAs, our data did not reproduce the high ROHF band as indicated in historical studies on single caner type. A possible explanation is that the ability of FRAs to induce ROH is different across cancer types. Since the inter-tissue ROH similarity heatmap shows a unique pattern of ROH in several cancer tissue types (Figure S4), thus probably only FRAs that can induce ROH across different cell lines will show association with high ROHF. However, no clear correlation could be observed at the genome-wide level between FRAs and high ROHF. This suggests that although FRAs can play a role in ROH formation, it is not the only cause of ROH formation. More interestingly, when comparing the NCI-60 to HapMap samples, the difference of ROHF was found lower in FRA regions than in non-FRA regions. These observations could be related to the incompleteness of current FRA database, or might suggest that FRAs could protect against ROH. In the latter case the FRA may function as a repair spot for restoration of accidentally mis-segregated chromatid during mitosis.

Pathway analysis on SNPs with higher ROHF in the adult versus young subgroups implicates a change in chemotaxis related genes. The genes in which these SNPs occur are marked with solid red circles in Figure 2. Currently it is not clear how ROH may qualitatively/quantitatively influence these genes. However, this observation is in consistent with a progression of metastatic tumor cells towards higher chemotaxis ability [33]–[35], which is correlated with their potential for invasion, intravasation, and metastasis and responsible for the attraction of carcinoma cells to blood vessels. The majority of the genes identified here, including but not limited to CCR1 [36], CCR2 [37], CCR3 [38], ENA78 [39], GPCR [40], GRO1 [41], GRO2/GRO3 [42], IP10 [43], IL-8 [44], NAP-2 [45], PF-4 [46] have been recently shown to associate with the progression of various types of cancer. We postulate that these genes might partly account for oncogenesis in the progression from low ROHF in healthy cell to high ROHF in cancer state. On the other hand, analysis of SNPs with high ROHF differences between the NCI-60 and HapMap samples shows a change in cell cycle and translation initiation related process, which might result from the progression of normal cells to cancerous cells towards autonomy and faster replication. Thus, these pathway analysis results may present a series of alterations leading to oncogenesis. However, additional evidence is still needed to fill the gap between the changes of genes in chemotaxis and cell cycle process, and the onset of oncogenesis.

The large number of ROH regions found in the NCI-60 cancer cell lines, and its significant association with the HapMap samples suggests a vital association of the ROH events with neoplastic progression. Importantly, the observation of higher ROHF in adults than in young, and the involvement of onco-related chemotaxis genes may provide a clue to understand the higher cancer incidence rate among older people. Together, these observations imply that the significant increase homozygotes in cancer cells might be a progression from changes started early in healthy state. Further investigation on the causal relationship between ROH and cancer will have to be conducted to shade light on the mechanisms leading to high ROH.

## Supporting Information

Figure S1 **NCI-60/HapMap ROHF correlation at genomic regions with different levels of HapMap ROHF.** The plot shows an increase in NCI-60/HapMap ROHF correlation coefficient (left axis, from 0.34 to 0.5), and NCI-60 ROHF (right axis, from 0.33 to 0.44) in genomic regions with increased levels of ROHF (from >0.1 to >0.95 quantile) in HapMap samples.
(TIF)

Figure S2 **Processes involved in SNPs with top ROHF differences.** Processes involved in SNPs with top ROHF difference between a) adult and young subgroups, and b) the NCI-60 and HapMap samples (see statistics section for details).
(TIF)

Figure S3 **Cell cycle G1_S phase.** Pathway analysis on SNPs with top ROHF difference between NCI-60 and HapMap show the involvement of Cell cycle G1_S phase. Red solid circles show genes covering SNPs with top change (see statistics section for details).
(TIF)

Figure S4 **Pair wise ROH similarity.** a) Intra tissue, b) Inter cell line (clustering based on pair-wise ROH similarities), and c) Inter tissue pair wise ROH similarity in the NCI-60 cancer cell lines
(TIF)

Table S1 **Genes in High ROHF bands without FRA in ±5 Mb vicinity (Regions identified in** **Table 3** **).**
(XLS)

Table S2 **Average upper 95th percentile ROHF and number of miR genes around FRAs.**
(XLS)

Table S3 **Genes with ROHF elevation in parent involved in chemotaxis process (Shown in** **Figure 2** **).**
(XLS)

Table S4 **Genes with high ROHF difference between NCI-60 and HapMap involved in cell cycle G1_S process.**
(XLS)

Table S5 **PLINK ROH module parameters setting.**
(XLS)
