# Supplementary figures and images for: Mass Homozygotes Accumulation in the NCI-60 Cancer Cell Lines As Compared to HapMap Trios, and Relation to Fragile Site Location
Source: PLoS One. 2012 Feb 9;7(2):e31628. doi: 10.1371/journal.pone.0031628 (PMC3276511; doi:10.1371/journal.pone.0031628)

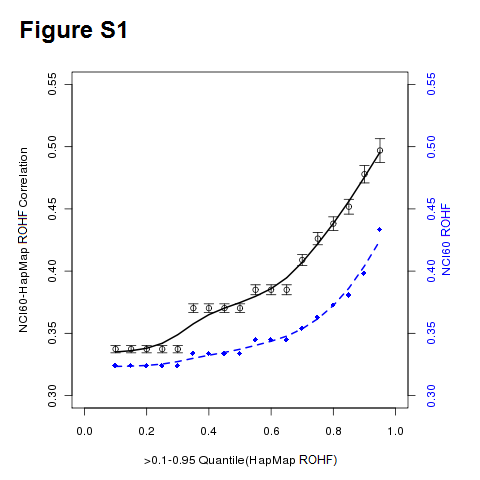

Supplement: Figure S1 — NCI-60/HapMap ROHF correlation at genomic regions with different levels of HapMap ROHF. The plot shows an increase in NCI-60/HapMap ROHF correlation coefficient (left axis, from 0.34 to 0.5), and NCI-60 ROHF (right axis, from 0.33 to 0.44) in genomic regions with increased levels of ROHF (from >0.1 to >0.95 quantile) in HapMap samples. (TIF) [file pone.0031628.s001.tif]

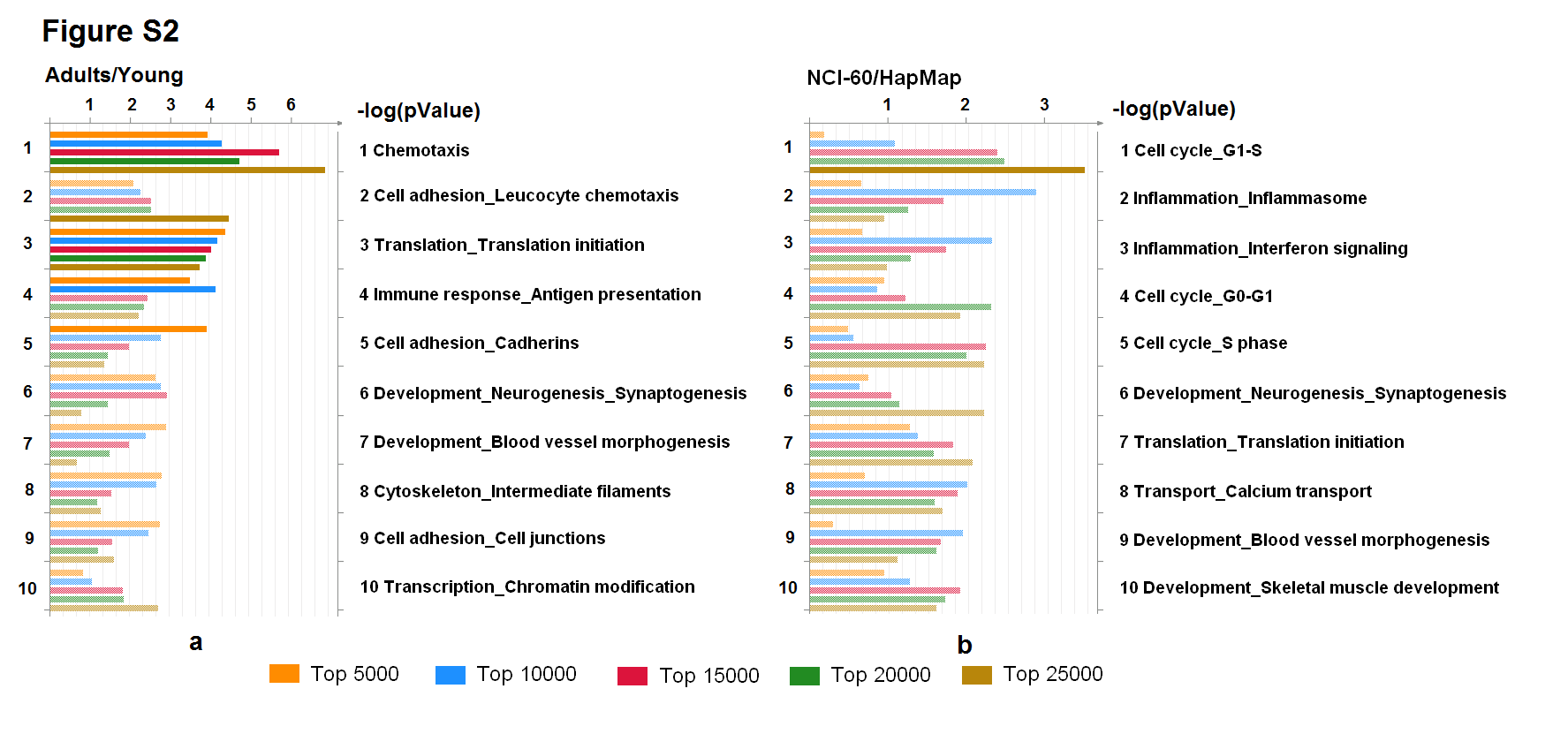

Supplement: Figure S2 — Processes involved in SNPs with top ROHF differences. Processes involved in SNPs with top ROHF difference between a) adult and young subgroups, and b) the NCI-60 and HapMap samples (see statistics section for details). (TIF) [file pone.0031628.s002.tif]

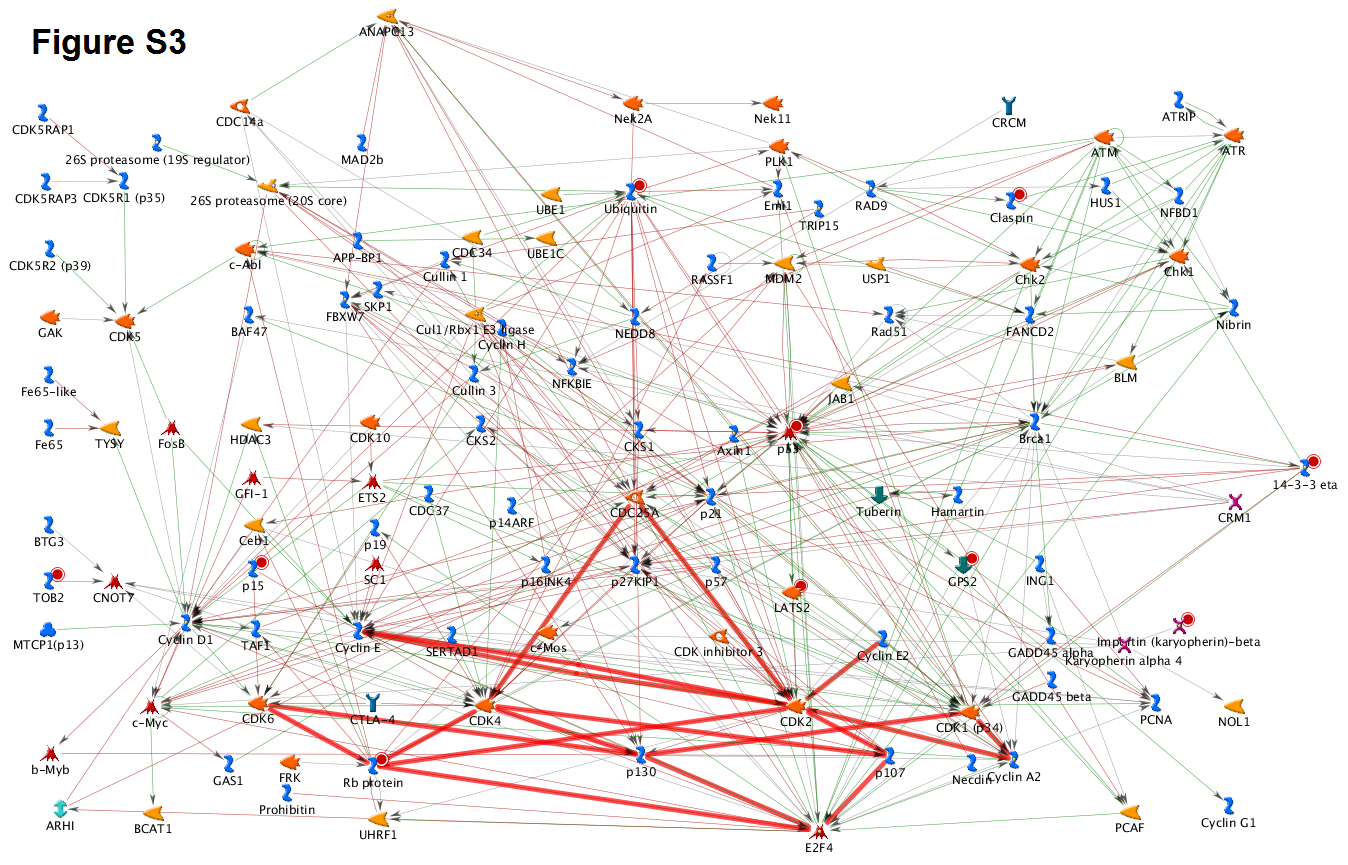

Supplement: Figure S3 — Cell cycle G1_S phase. Pathway analysis on SNPs with top ROHF difference between NCI-60 and HapMap show the involvement of Cell cycle G1_S phase. Red solid circles show genes covering SNPs with top change (see statistics section for details). (TIF) [file pone.0031628.s003.tif]

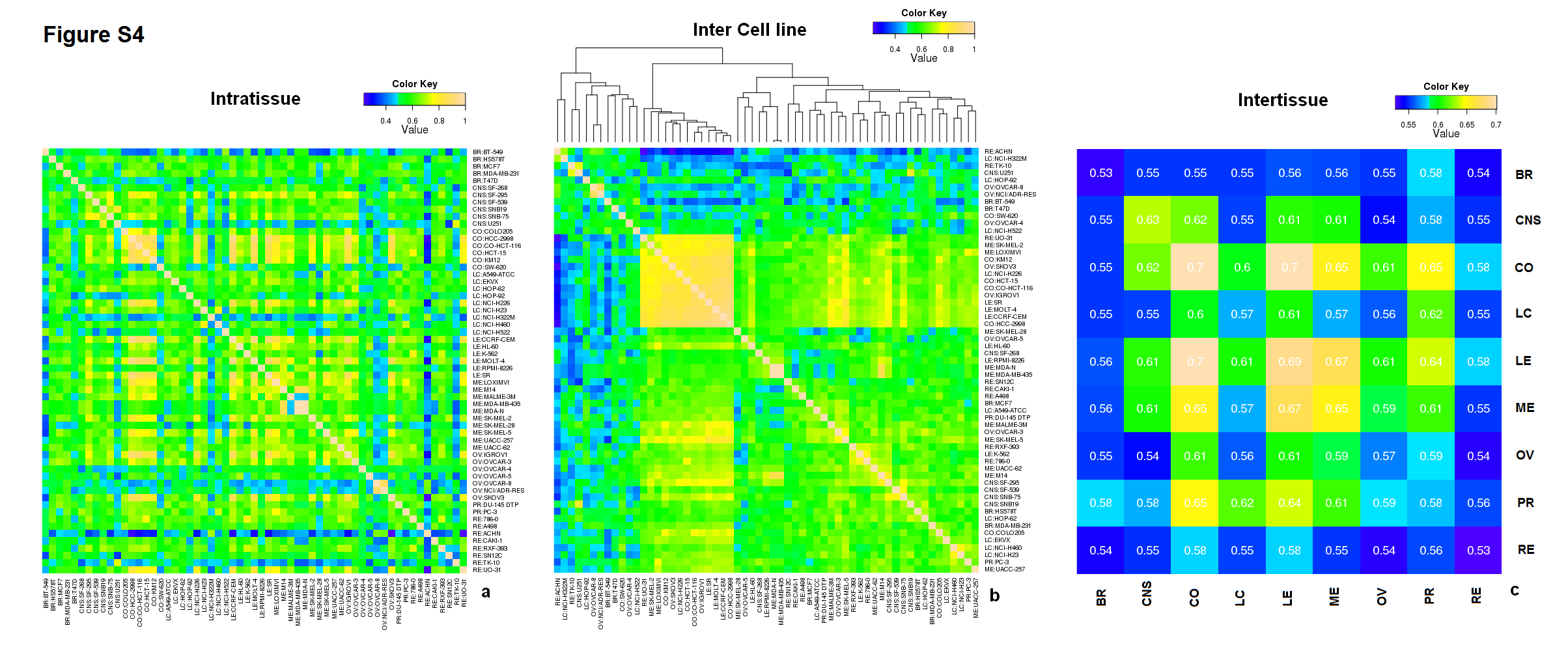

Supplement: Figure S4 — Pair wise ROH similarity. a) Intra tissue, b) Inter cell line (clustering based on pair-wise ROH similarities), and c) Inter tissue pair wise ROH similarity in the NCI-60 cancer cell lines (TIF) [file pone.0031628.s004.tif]
